# Supplementary material for: Splice-Junction-Based Mapping of Alternative Isoforms in the Human Proteome
Source: Cell Rep. Author manuscript; Available in PMC 2020 Jan 15. (PMC6961840; doi:10.1016/j.celrep.2019.11.026)
Supplement: 2 [file NIHMS1546469-supplement-2.zip › DF1/PXD009737/TestisLysC/TestisLysC_1_SPTAN1_LRQEFAQHANAFHQWIQETRSCMVEESGTLESQLEATK.pdf]

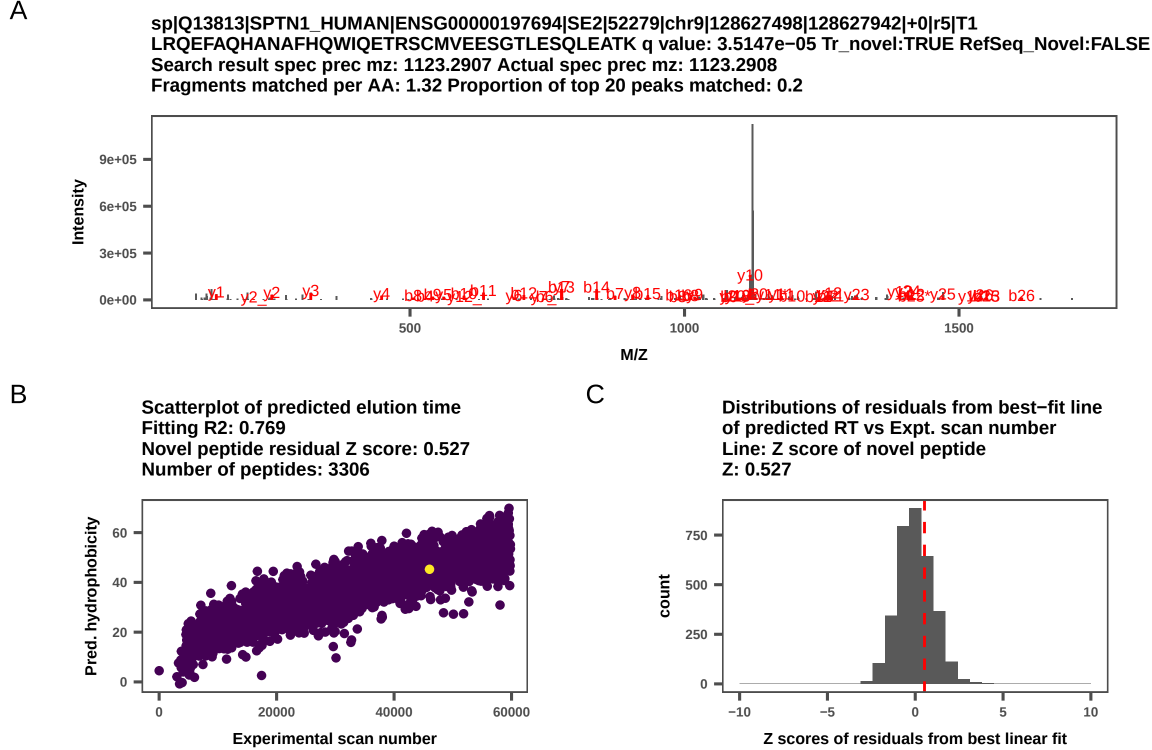
A
sp|Q13813|SPTN1_HUMAN|ENSG00000197694|SE2|52279|chr9|128627498|128627942|+0|r5|T1
LRQEFAQHANAFHQWIQETRSCMVEESGTLESQLEATK q value: 3.5147e−05 Tr_novel:TRUE RefSeq_Novel:FALSE
Search result spec prec mz: 1123.2907 Actual spec prec mz: 1123.2908
Fragments matched per AA: 1.32 Proportion of top 20 peaks matched: 0.2
Intensity
9e+05
6e+05
3e+05
0e+00
y1
y2
y3
y4
b11
b14
y10
y23
y25
b26
500
1000
1500
M/Z
B
Scatterplot of predicted elution time
Fitting R2: 0.769
Novel peptide residual Z score: 0.527
Number of peptides: 3306
Pred. hydrophobicity
60
40
20
0
0
20000
40000
60000
Experimental scan number
C
Distributions of residuals from best−fit line
of predicted RT vs Expt. scan number
Line: Z score of novel peptide
Z: 0.527
count
750
500
250
0
−10
−5
0
5
10
Z scores of residuals from best linear fit
